# Oil of Cloves to Remove the Antipathy against Chloroform

**Published:** 1884-12

**Authors:** 


### Oil of Cloves to Remove the Antipathy Against Chloroform.

Not at all unfrequently patients are met with who have an aversion against the odor of chloroform to such an extent that it cannot be administered to them with any degree of satisfaction. The addition of some of the corrective ethereal oils seldom acts satifactorily. The best remedy, in universal use in England at present is the addition of a little sulphuric ether and ethereal oil of cloves to the chloroform. Nussbaum employs this mixture. The odor is agreeable to most of the patients. Nussbaum places a great deal of stress on the ability of being able to narcotize a patient well, therefore his saying: "It is more difficult to narcotize well than to operate well." Of the 150 cases tabulated by Krod of death from chloroform narcosis a large number occurred during the stage of excitation, for which the physician in every instance should be made responsible, while if death should occur during tolerance he cannot be held accountable.—*From Memorabilien.*

A CURIOUS case of death from the bite of a pig has been reported in Birmingham. The son of a pork-butcher, while playing with a pig, was bitten on the hand. The case was treated at a hospital, but symptoms of blood-poisoning supervened, and the patient died. No further details are given, nor does the report mention if the blood-poisoning was regarded as due directly to the bite, or was of secondary nature. There is nothing specially dangerous about the bite of a pig, but the explanation of the above case may possibly be found to rest more upon the recognition of the foul habits of the animal occasioning a poisoned wound, than upon anything "poisonous" in the pig's bite by itself. A clean pig's bite, in other words, is not dangerous in so far as poisoning the wound is concerned, any more than would be the bite of a man, though it is possible that where the teeth or mouth-secretions are infected in either case mischief might follow. —*Health.*

